# Transgenic technologies to induce sterility

**DOI:** 10.1186/1475-2875-8-S2-S7

**Published:** 2009-11-16

**Authors:** Flaminia Catteruccia, Andrea Crisanti, Ernst A Wimmer

**Affiliations:** 1 Imperial College London, Division of Cell and Molecular Biology, Imperial College Road, London SW7 2AZ, UK; 2 Georg-August-University Göttingen, Johann-Friedrich-Blumenbach-Institute of Zoology and Anthropology, Dept. Developmental Biology, GZMB, Ernst-Caspari-Haus, Justus-von-Liebig-Weg 11, 37077 Göttingen, Germany

## Abstract

The last few years have witnessed a considerable expansion in the number of tools available to perform molecular and genetic studies on the genome of *Anopheles *mosquitoes, the vectors of human malaria. As a consequence, knowledge of aspects of the biology of mosquitoes, such as immunity, reproduction and behaviour, that are relevant to their ability to transmit disease is rapidly increasing, and could be translated into concrete benefits for malaria control strategies. Amongst the most important scientific advances, the development of transgenic technologies for *Anopheles *mosquitoes provides a crucial opportunity to improve current vector control measures or design novel ones. In particular, the use of genetic modification of the mosquito genome could provide for a more effective deployment of the sterile insect technique (SIT) against vector populations in the field. Currently, SIT relies on the release of radiation sterilized males, which compete with wild males for mating with wild females. The induction of sterility in males through the genetic manipulation of the mosquito genome, already achieved in a number of other insect species, could eliminate the need for radiation and increase the efficiency of SIT-based strategies. This paper provides an overview of the mechanisms already in use for inducing sterility by transgenesis in *Drosophila *and other insects, and speculates on possible ways to apply similar approaches to *Anopheles *mosquitoes.

## Background

Vector-borne diseases cause a considerable burden for human health in tropical and subtropical regions. Malaria alone, transmitted exclusively by *Anopheles *mosquitoes infected with *Plasmodium *protozoan parasites, causes the death of more than a million people each year, most of which occur in sub-Saharan Africa. Traditional strategies aimed at tackling malaria have often focused on reducing human-mosquito contact with bednets and the suppression of vector populations, principally through the use of insecticides. However, the rapid appearance of insecticide resistance in vector species is hampering the eradication of this devastating disease, and efforts aimed at developing novel and more effective malaria control strategies are intensifying.

Amongst the novel approaches, the use of transgenesis, until a few years ago just a remote idea [1,2], has recently gained considerable attention. Techniques for the genetic modification of a number of anopheline species that transmit malaria (*Anopheles stephensi*, *Anopheles gambiae *and *Anopheles albimanus*) have now been developed [3-5] and provide the means to manipulate the ability of mosquitoes to serve as disease vectors. In the case of the major vector of human malaria, *An. gambiae*, germline transformation, even if still technically challenging, is achieved at reasonable frequencies and, despite the limited number of publications on the subject, is carried out in a number of laboratories [6-8]. The potential of a transgenic approach for the control of this important vector species is tremendously strengthened by the availability of genetic and genomic tools (such as RNA interference techniques, microarray platforms, and the full genome sequence) essential to perform functional studies on specific genes and to analyse different aspects of mosquito biology at the organismal level [9-12]. In recent years studies on the factors and mechanisms that shape vectorial capacity [13-16] and the interactions between the vector and the parasite [17-19] have intensified, and a clearer understanding of the genetic bases of mosquito biology and behaviour is starting to emerge. In this scenario of rapid technological progress and crucial advancement in understanding of both the vector and the parasite biological systems, transgenic technologies are being proposed to support, replace, or complement traditional vector control methods.

One approach involves the introgression of factors interfering with parasite development into field populations of vectors. Theoretically, natural disease vectors could be replaced with genetically modified anopheline mosquitoes that are rendered refractory to the transmission of malaria parasites. The merits of such a control strategy, known as population replacement, are demonstrated by the various levels of refractoriness to the transmission of murine malaria, *Plasmodium berghei*, achieved in the laboratory by the expression of a series of effector genes [6,7]. Intense efforts are being focused on the search for factors that can act on parasites relevant to human health, as well as for effective technologies to drive such factors through field populations. A second approach for the use of transgenic technologies is focused on improving the implementation of the sterile insect technique (SIT) [20,21] or its transgenic derivative, the release of insects carrying a dominant lethal genetic system (RIDL) [22-24]. As thoroughly discussed elsewhere in this supplement [25], SIT is a species-specific, environmentally friendly strategy for the control of major insect pests which relies on the repeated release of sterilized insects (i.e. not capable of producing viable progeny upon mating) over large areas to achieve suppression or eradication of field populations. The use of transgenic technologies can improve SIT for anophelines by helping to solve three major issues: reduced male competitiveness due to the sterilization procedure (irradiation or chemosterilisation) [26]; the need for genetic sexing strains so as to release only males [27,28]; and the need to monitor the survival and dispersal of the sterile males in the field. In this paper, an update on the use of transgenic technologies to induce sterility using embryonic or late stage lethality in *Anopheles *males will be provided, and possible strategies for achieving this using *Drosophila melanogaster *as the paradigm will be discussed. Other methods currently discussed for achieving what is in effect conditional sterility in insect progeny (such as the use of inherited dominant lethals [29]) are not reviewed here.

## The advantages of using transgenic sterility over irradiation

Currently, reproductive sterility in insects to be used in SIT programmes is induced following their exposure to gamma radiation from a ^60^Co or ^137^Cs source. Chemosterilization has also been used in the past, especially for mosquitoes, however there is a laboratory report of non-target sterility [30] causing concern for the environment and for human health (but see [31,32] for more details). Radiation induces chromosomal damage in the germ cells, leading to the desired sterility [32] but also causes deleterious somatic effects that reduce the competitiveness of the males. Thus a balance has to be struck between the radiation dose, the induced sterility, the competitiveness of the male and the final level of reproductive sterility induced in the wild females [33]. Quantifying the negative effects of radiation on sterile male competitiveness has produced extremely divergent figures in the literature. For example in the Mediterranean fruit fly *Ceratitis capitata*, one study showed a 10-fold reduction in competitiveness [34] whilst another could demonstrate no negative effect of radiation [35]. Radiation is also usually carried out at the life stage which is most convenient logistically, i.e. the pupal stage, and not at the stage most optimal biologically, i.e. the adult stage. In *An. gambiae*, a recent study has demonstrated that irradiating pupae, although more practical, is associated with reduced mating competitiveness of the resulting adults [26] (however see [36] for *An. arabiensis*). Adult irradiation would, therefore, be the best option, but it is associated with obvious logistical difficulties.

By eliminating radiation, insect competitiveness would be improved and the insects could be released at any stage of development. The logistics involved with the radiation procedure would disappear, there would be one less deleterious treatment for the released insects, and there would be no need for an expensive and politically problematic radiation source, although the recent development of new X-ray machines should deal with this issue.

**Figure 1:**
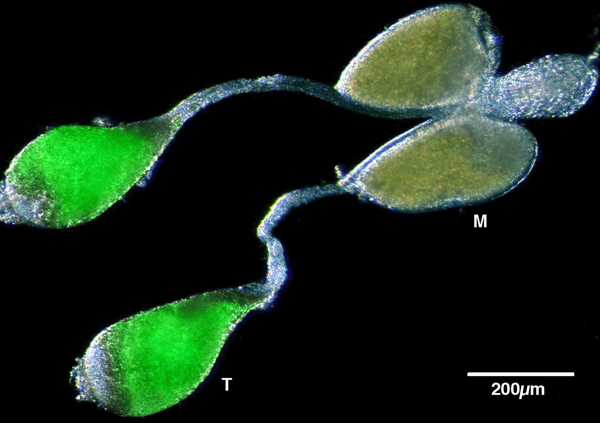
**The internal reproductive organs of a male *Anopheles gambiae *transgenic mosquito**. The male accessory glands (M), in which seminal secretions containing proteins and peptides are produced, and the testes (T), where sperm cells develop, are indicated. The image is an overlay of a fluorescent and a transmission microphotograph. The fluorescence in the testes is derived from the expression of a green fluorescent protein *egfp *reporter gene driven by the testis-specific β*2-tubulin *promoter [37]. The scale bar is indicated.

It may also be possible to combine transgenic sterility induction with a genetic sexing mechanism to produce populations of male-only sterile mosquitoes. Transgenic sexing strains have been developed in *An. stephensi*, a major vector for human malaria in Asia, which can achieve reliable separation of male individuals at an early developmental stage based on the expression of a fluorescent marker in sperm cells driven by a testis-specific *β2-tubulin *promoter (Figure 1). Reliable sex separation has also been achieved in these lines using non-destructive automated larval sorters [37]. In principle, such sperm-specific markers could be linked to the factor(s) inducing sterility, thereby providing the possibility to meet three highly desirable objectives: 1) the automated separation of males from females at the larval stage, 2) the monitoring of mating of sterile males with wild females through the identification of fluorescent spermatozoa in the spermatheca, the female sperm storage organ, and 3) the prevention of the production of viable progeny. The development and testing of constructs combining genetic sexing (see [38] for genetic sexing mechanisms) and sterility is a research priority for future studies aimed at improving sterility strategies.

## How could sterility be induced in *Anopheles*?

In order for SIT programmes to be successful, males must be effective at preventing the females they mate with from reproducing. This relies on males being able to transfer 1) sterile sperm to ensure death of developing zygotes and 2) accessory gland secretions to induce the appropriate behavioural responses in the female. These behavioural responses include prevention of remating. *Anopheles gambiae *females mate only once in their lifetime and after a successful copulation a life-long refractoriness to further mating is induced. In many insects, it has been unambiguously established that reduction or complete suppression of mating responsiveness is due to the transfer of factors (mainly proteins and peptides) from the male accessory glands during copulation, with sperm also playing an important role [39-42]. Although the triggers of this post-mating response have not been identified with certainty in *Anopheles *[43-46], it is likely that analogous mechanisms are in place. Indeed, recently a large number of genes and proteins with homology to important *Drosophila *male accessory gland proteins have been identified in *An. gambiae *[47] (see Figure 1 for an image of the male reproductive tract including testes and male accessory glands dissected from an *An. gambiae *male). Control strategies based on the use of transgenesis to induce sterility must take into consideration the effects it will have on the ability of the males to induce normal post-copulatory responses in females. In order to gain crucial insights on how field populations will respond to any intervention, it will be essential to intensify research efforts to understand the genetic and physiological bases of reproduction in *Anopheles *mosquitoes.

In the next four sections, the mechanisms used in *Drosophila *and other higher dipterans to induce reproductive sterility will be discussed as will potential approaches on how such mechanisms could be transferred and adapted to *Anopheles *mosquitoes.

## Reproductive sterility based on lethality of the progeny

An elegant strategy to develop sterile males is based on the expression of a dominant conditional lethal factor. However, it will be very important that the lethal system is inactive during spermatogenesis and in adult males and that the expression of the construct can be repressed under permissive rearing conditions in the facility. After release, non-permissive conditions will not affect spermatogenesis or the adult males themselves but will result in death of their progeny. If a male is homozygous for such a gene construct, each fertilization event will lead to lethality in the progeny.

Lethality could be induced by a number of cytotoxic gene products driven by suitable promoters. The use of polypeptide toxins, such as the diphtheria or ricin, is probably not suitable as mosquitoes expressing these proteins may not be perceived favorably by regulatory agencies. However, dominant negative protein variants that cause cytotoxicity, such as the constitutively activated cell signaling molecule Ras64B, have been shown to cause organismal lethality in *Drosophila *[23] and might provoke less concern. The products of proapoptotic genes that induce apoptosis of the cells in which they are expressed represent another possible choice. In *Drosophila*, three proapoptotic genes *reaper *(*rpr*), *grim*, and *head involution defective *(*hid*) have been identified [48,49]. After conditional activation of these genes, ectopic apoptosis is induced which causes organismal lethality. Phylogenetically conserved activity has been shown for all three genes, as apoptosis can be induced by *rpr *in a lepidopteran cell line [50] and the frog *Xenopus laevis *[51], as well as by *grim *and *hid *in mammalian cell lines [52,53]. Thus, systems based on these *Drosophila *proapoptotic effector genes should be transferable to other species including disease vectors. To enhance the strength of the lethality effect, a constitutive active form of *hid *that cannot be down-regulated by cell signaling pathways can also be used [54].

Since a lethal strain would be impossible to maintain, the expression of lethality needs to be conditional. This can be achieved by using a two-component system, which will be repressed during rearing due to an additive in the diet but which, in the absence of the additives in the field, will be expressed. One component of the system will be a "driver" construct that will express a conditional mediator under the control of a suitable promoter. The second component will be an "effector" construct that reacts to the mediator, depending on the presence or absence of the additive, and will lead to the expression of cytotoxic genes. Before release, the strains will be switched to additive-free food and the two-component system will turn on and cause the dominant sterility in the released males based on lethality of their progeny. For this purpose, the tetracycline-controllable transactivator (tTA) system [55] can be used. The hybrid transactivator tTA represents a fusion between the bacterial Tet-repressor and the *Herpes simplex *VP16 activation domain. In the presence of tetracycline the tTA cannot bind DNA and the system is switched "off". However, once the tetracycline is removed from the diet or when the strain is released, tTA will bind to the DNA at Tet responsible elements (TRE) and activate transcription of effector constructs under the control of TRE sites. The tTA/TRE system is extremely tight: even the expression of potent toxin genes is sufficiently repressed when tetracycline is provided. This system has already been shown to be able to control gene expression in *An*. *stephensi *[56].

## Embryonic lethality: the proof of principle in *D. melanogaster*

One way to achieve control of a lethal system to avoid affecting spermatogenesis and adult males would be to use a promoter that is active at early blastoderm stages only. Such a transgene-based embryo-specific lethality system was successfully tested in *Drosophila *and generated reproductively sterile males [57]. The system was based on the ectopic expression of the hyperactive phospho-acceptor-site mutant allele *hid*^*Ala*5 ^[54], which caused lethality when driven by the tetracycline-controlled transactivator tTA. To ensure early embryonic expression, tTA was regulated by an enhancer/promoter from the cellularization genes *nullo *or *serendipity α *(*sry α*) [57]. In order to restrict the detrimental effects of a dominant lethality system to the embryo, *cis*-regulatory elements that are active during the earliest possible stages of embryogenesis and whose activity is entirely confined to these stages are of utmost importance. The *D. melanogaster *cellularization genes *sry α *and *nullo *encode structural components of the microfilament network that are required for blastoderm cellularization and are expressed specifically during the insect-typical superficial cleavage stages. Thus their strong and ubiquitous but stage-specific expression seemed ideal to drive an embryo-specific lethality system. This transgenic approach caused reproductive sterility that neither interfered with the adult phase of the insect life cycle nor with spermatogenesis and generated competitive males that can transfer sperm [57].

## Embryonic lethality: transfer to other insects

Because HID and tTA function not only in *Drosophila *but also in mammalian systems and mosquitoes, the question of transferability of the *Drosophila *proof-of-principle [57] was mostly concerned with the functional conservation of the *cis*-regulatory control elements of the cellularization genes. A direct transfer of the *Drosophila *construct using the *sry *α promoter to drive *tTA *expression to the Mediterranean fruit fly yielded transgenic flies that did not show any expression of *tTA *[58]. This indicates that the cellularization specific *sry *α promoter from *Drosophila *is not functional in the Mediterranean fruit fly despite the relative close phylogenetic relationship of these cyclorrhaphan flies. Thus in order to get functional promoters, endogenous promoters of Mediterranean fruit fly had to be isolated. A first approach to obtain homologues of the genes *nullo *and *sry α *using degenerate primers was unsuccessful as these cellularization genes are evolving too rapidly [59] and a cDNA subtraction approach was used. The time window of cellularization in Mediterranean fruit fly was first determined and genes were isolated that were specifically expressed during the superficial cleavage stages [59]. The promoter/enhancer regions of these genes were then isolated by inverse PCR. These promoter/enhancer elements of cellularization-specifically expressed genes were subsequently used to generate a reproductive sterility system for this species based on transgenic embryonic lethality [59]. These elements act differently in expression strength and in their ability to drive lethal effector gene activation and they are strongly influenced by position effects. However, out of several combinations of driver and effector construct integrations, several resulted in larval and pupal lethality and one combination (LL#67) showing complete embryonic lethality. Since the transgenic strain LL#67, appeared to be competitive with wild-type flies in laboratory and field cage tests, it might ultimately be possible to use it, or a similar strain, to improve the efficacy of operational Mediterranean fruit fly SIT programmes. This successful transfer of the *Drosophila *proof-of principle embryonic lethality system represents a straightforward approach that should also be applicable to anopheline malaria vectors.

Ideally, such an embryonic lethality system should be combined with a genetic sexing system causing conditional female lethality so that vigorous, reproductively sterile male mosquitoes could be produced for the use in SIT programmes. In anopheline mosquitoes only one transgenic sexing systems has so far been produced [37], and alternative approaches could be modelled after systems that use either female specific promoters as shown in *Drosophila *[23,60], which however might act too late for effective mass production, or female specifically spliced introns as shown for Mediterranean fruit fly [61].

## Postembryonic lethality

Inducing lethality at later stages could be advantageous for mosquitoes as transgenic larvae would compete with wild larvae for resources under density dependent conditions in the field [62]. To achieve this, a simplified one-component system based on the toxicity of the tTA transactivator itself when expressed at high levels has been developed and tested in the Mediterranean fruit fly [63]. In this system, expression of the tTA transactivator is directly controlled by the TRE: in the presence of tetracycline, tTA is inactivated and, therefore, expressed at basal levels, while when tetracycline is removed from the diet, the basal expression of tTA induces an autoregulatory loop that causes the synthesis of more tTA, which accumulates to high levels inducing lethality. When heterozygous males were crossed to wild type females, half the progeny reared in a non-tetracycline diet generally died, as expected. This system induced lethality in Mediterranean fruit fly at later developmental (larval and pupal) stages with a number of adult escapers recovered.

The same tetracycline-repressible dominant lethal system was also used in the yellow fever and dengue vector *Aedes aegypti *to induce killing of both male and female mosquitoes [62]. A number of transgenic lines were developed and reared in the presence and absence of tetracycline in their diet. The lethality trait was highly penetrant, however as seen in the case of Mediterranean fruit fly, a small proportion of transgenics survived to adulthood in the absence of the drug. It is reasonable to envisage that it will be possible to optimize this system by adding an additional lethal factor to achieve complete penetrance of the lethality trait. This will be necessary to avoid an unwanted ingression of adult transgenic individuals into wild mosquito populations that would interfere with monitoring of the SIT programme and could become the basis for resistance development.

## *Anopheles *mosquitoes: current perspectives for inducing male sterility

The examples provided in the previous sections show how reproductive sterility based on embryonic or postembryonic lethality can be induced by transgenic means in insects other than *D. melanogaster*. As mentioned above, the use of the tTA transactivator system for conditional gene expression has already been demonstrated in *An. stephensi *[56], and there is no reason to doubt that the one-component system will also be capable of inducing lethality in *An. gambiae*. However, other avenues for inducing male sterility or embryonic lethality are worth pursuing.

Recently, a *reaper*/*grim*-like proapoptotic gene has been identified in the *An. gambiae *genome. This gene, named *michelob_x *(*mx*), despite not coding for a Grim helix 3 (GH3) domain, was capable of inducing cell death in *D. melanogaster *S2 and *Aedes albopictus *C6/36 cell lines within 20 h of transfection [64]. The apoptotic activity was shown to be dependent on the N-terminal IAP (inhibitor of apoptosis protein)-binding motif of the molecule, whose removal completely abolished killing. In the same study, *mx *also induced embryonic lethality *in vivo *in the progeny of the crosses of transgenic *D. melanogaster *expressing a UAS-mx fusion in a specific set of cells of the central nervous system and the ventral epidermis driven by a *P52Gal4 *insertion [65]. Although it is possible that such lethality was due to a 'leakiness' of the *P52Gal4 *insertion, as a parallel anti-β-galactosidase staining showed that many epithelial cells were also killed by *mx *expression, these experiments demonstrate the ability of *mx *from *An. gambiae *to function as a potent proapoptotic gene in an unrelated species.

A novel mechanism has recently been proposed to create sex ratio distortions and embryonic lethality in natural populations based on the use of a homing endonuclease enzyme [8]. Sex ratio distortions have been described in *Ae. aegypti *and *Culex pipiens *mosquitoes in which Y chromosomes are naturally driven in field populations probably through the induction of breaks on the X chromosome during male meiosis [66]. Recently there has been renewed interest in using this system for population replacement in *Ae. aegypti *[67,68]. A similar system could be artificially created in *An. gambiae *using the property of the I-*Ppo*I homing endonuclease: this enzyme has been recently shown to be able to cleave chromosomal rDNA repeats in the *An. gambiae *Sua 4.0 cell line, leading to cell proliferation arrest and possibly to cell death [8]. As in *An. gambiae *and other anopheline species the rDNA region is only present in the centromeric region of the X chromosome, expressing I-*Ppo*I in the sperm cells during male meiosis could potentially produce incapacitated X-carrying spermatozoa. Recently, transgenic *An. gambiae *lines were developed which express I-*Ppo*I under the control of the β*2-tubulin *promoter. Expression of I-*Ppo*I in the testes induced a strong bias toward Y chromosome-carrying spermatozoa, and also induced complete early dominant embryo lethality in crosses with wild-type females. Embryos originating from transgenic males were arrested very early in their development, probably at a point before the fusion of the male and the female pronuclei [69].

In parallel to the quest for potent effector genes and mechanisms capable of inducing sterility, there is the need to identify suitable promoters to drive their expression. This is a considerable limitation of the system in *Anopheles*, as besides the β*2-tubulin *regulatory regions described above [37] (Figure 1), which are selectively transcribed at the mature primary spermatocyte stage just before meiotic division starts, no other male or female germline-specific promoter has been successfully characterized and used in transgenic individuals. Possibly, early cellularization promoters similar to those utilized in *Drosophila *and in Mediterranean fruit fly could be identified as outlined above and used to transcribe toxic genes during embryogenesis, thereby mediating reproductive sterility. It is reasonable to speculate that in the near future it will be possible not only to adapt existing strategies but also to develop novel, possibly species-specific ones, to create populations of transgenic *Anopheles *males that can be used in release programmes.

## Conclusion

The development and adaptation of transgenic technologies to anopheline vectors of human malaria represents an important tool available to the mosquito research community. Beyond its importance for performing functional studies, genetic manipulation of the mosquito germline can be exploited to support and complement vector control strategies such as those based on conventional SIT. The induction of sterility by transgenic means could undoubtedly provide important advantages to SIT programmes by eliminating the need for radiation. The examples available from other insects encourage optimism: a number of mechanisms are available to be tested in anopheline vectors for their efficacy and safety. Additional strategies such as those proposed here could be attempted that may have the advantage of a species-specific application. The possibility of combining sterility with a sexing mechanism will render genetic manipulation of the vector a crucial tool for a successful deployment of SIT strategies targeting anopheline populations in the field.

## Competing interests

EAW declares competing financial interests, as the Georg-August-University Göttingen has filed a patent application on key inventions partially described in this manuscript. The patent application is titled: "Developmental stage-specific lethality system for insect population control". The other authors declare no competing financial interests.

## Authors' contributions

FC designed the paper, FC, AC and EAW wrote it.
